# Investigation of the Role of the Spike Protein in Reversing the Virulence of the Highly Virulent Taiwan Porcine Epidemic Diarrhea Virus Pintung 52 Strains and Its Attenuated Counterpart

**DOI:** 10.3390/v12010041

**Published:** 2019-12-30

**Authors:** Chi-Fei Kao, Hui-Wen Chang

**Affiliations:** 1 School of Veterinary Medicine, National Taiwan University, Taipei 10617, Taiwan; fei81005@gmail.com; 2 Graduate Institute of Molecular and Comparative Pathobiology, School of Veterinary Medicine, National Taiwan University, Taipei 10617, Taiwan

**Keywords:** PEDV, reverse genetics, spike protein, attenuation, virulent determinant

## Abstract

Porcine epidemic diarrhea virus (PEDV) has continuously caused severe economic losses to the global swine industries; however, no successful vaccine against PEDV has been developed. In this study, we generated four autologous recombinant viruses, including the highly virulent iPEDVPT-P5, attenuated iPEDVPT-P96, and two chimeric viruses (iPEDVPT-P5-96S and iPEDVPT-P96-5S) with the reciprocally exchanged *spike* (*S*) gene, to study the role of the *S* gene in PEDV pathogenesis. A deeper understanding of PEDV attenuation will aid in the rational design of a live attenuated vaccine (LAV) using reverse genetics system. Our results showed that replacing the *S* gene from the highly virulent iPEDVPT-P5 led to complete restoration of virulence of the attenuated iPEDVPT-P96, with nearly identical viral shedding, diarrhea pattern, and mortality rate as the parental iPEDVPT-P5. In contrast, substitution of the *S* gene with that from the attenuated iPEDVPT-P96 resulted in partial attenuation of iPEDVPT-P5, exhibiting similar viral shedding and diarrhea patterns as the parental iPEDVPT-P96 with slightly severe histological lesions and higher mortality rate. Collectively, our data confirmed that the attenuation of the PEDVPT-P96 virus is primarily attributed to mutations in the *S* gene. However, mutation in *S* gene alone could not fully attenuate the virulence of iPEDVPT-P5. Gene (s) other than *S* gene might also play a role in determining virulence.

## 1. Introduction

Porcine epidemic diarrhea virus (PEDV) is an enveloped, positive-sense and single stranded RNA virus belonging to the genus *Alphacoronavirus*. PEDV is the causative agent of porcine epidemic diarrhea (PED), a historic, highly contagious enteric swine disease characterized by diarrhea, dehydration and poor growth performance in pigs at all ages [1,2]. PED was first identified in 1971 in the United Kingdom, and subsequently became an endemic disease in the Europe and most Asian countries with a low mortality rate and limited economic losses [1]. For decades, the disease was controlled through the use of live attenuated vaccines (LAVs) derived from the PEDV prototypes, CV777- or DR13. Unfortunately, in late 2010, a new highly virulent PEDV strain emerged in China and quickly spread worldwide, including Taiwan [3,4]. Moreover, the conventional LAV failed to induce protection against this new virulent PEDV strain, consequently resulting in nearly 100% mortality amongst neonatal piglets and leading to substantial economic impacts on swine markets in the affected regions [5]. Phylogenetic analysis categorizes PEDV into two genogroups [2]. Genogroup I consists primarily of the historic, low-virulent PEDV strains that appeared around 1970, whereas genogroup II is composed of the highly virulent PEDV strains, which emerged after 2010s. Both genogroups can be further divided into two subgroups each, namely G1a, G1b, G2a and G2b though a recent phylogenetic study identified a novel recombinant PEDV strain classified into a new G1c clade [6].

The complete genome of PEDV is approximately 28 kilobase pairs and consists of seven open reading frames (ORF). Out of the seven, four ORFs are responsible for encoding structural proteins, namely the spike (S) glycoprotein as well as envelop (E), membrane (M), and nucleocapsid (N) proteins, which are crucial for viral morphogenesis and establishment of infection establishment [7]. Among all coronaviruses, S glycoprotein plays a particularly essential role in cell-virus interaction and acts as the vital determinants of viral virulence/attenuation [8,9,10]. S glycoprotein is a homotrimeric type I fusion protein, which can be cleaved into two parts—the S1 and S2 subunits—by cellular protease [11]. The S1 subunit contains the receptor binding domain (RBD) and several neutralizing epitopes that are essential for determining host and tissue tropism, and triggering protective host immunity, respectively. Therefore, the S1 subunit has long been considered to be the primary target for vaccine development. The S2 domain, on the other hand, mediates membrane fusion which facilitates release of viral RNA and infection to the neighboring cells [8].

LAVs have been shown to be the most effective way to elicit protective immunity against PEDV infection [12]. Therefore, to combat the emergence of PED as well as to immediately suppress any future outbreaks, studies have been carried out to develop safe and effective vaccines [13,14]. Aside from serial cell culture or animal passages, the reverse genetic system, which rescues genetically modified attenuated viruses, is now the common approach in generating LAV candidates for both human and animal viral diseases, including PEDV [15,16]. To this end, understanding the molecular biology and identification of virulent/attenuating determinant(s) are necessary for the rational design of potential LAV candidates. Comparison of the genomic differences between the Vero cell-cultured attenuated G2b PEDV and their corresponding parental virulent strains has revealed a common pattern of mutation that particularly affects the *S* gene [12]. While the observation is reasonable considering the fundamental role of S protein in PEDV infection and induction of host immunity, a recent publication found that a singular *S* gene exchange had no influence on the virulence of the highly virulent G2b BJ2011C strain and avirulent G1a CHM2013 strain [17].

In the present study, we utilized reverse genetics to examine the role of the *S* gene in the attenuation process of PEDV using the highly virulent and attenuated G2b PEDV Pintung (PEDVPT) 52 strains, the PEDVPT-P5 and PEDVPT-P96 viruses. Nonetheless, our findings were discordant with previous reported data [17]. We found that replacement of the *S* gene with the iPEDVPT-P5 virus enabled iPEDVPT-P96 to regain its virulence. A reciprocal approach revealed that iPEDVPT-P5 virus became partly attenuated after the *S* gene was exchanged to iPEDVPT-P96. Collectively, we concluded that the *S* gene is of critical importance to the attenuation process of the PEDVPT 52 strain. However, mutation in the *S* gene alone could not completely attenuate the iPEDVPT-P5 virus. Thus, gene(s) other than *S* gene could also play a role in determining the virulence.

## 2. Materials and Methods

### 2.1. Ethics Statement

All procedures involving animal experiment were reviewed, approved and conducted in strict accordance with the Institutional Animal Care and Use Committee (IACUC) of National Taiwan University (Taiwan, Republic of China) with the approval No.: NTU105EL-00160.

### 2.2. Cells and Viruses

Vero C1008 cells (ATCC No. CRL-1586) were maintained in growth medium containing Dulbecco’s modified Eagle’s medium (DMEM, Gibco, Grand Island, NY, USA) supplemented with 10% fetal bovine protein (FBS), 250 ng/mL Amphotericin B, 100 U/mL Penicillin and 100 μg/mL Streptomycin. The recombinant viruses (iPEDVPT-P5 and iPEDVPT-P96), and the chimeric viruses (iPEDVPT-P5-96S and iPEDVPT-P96-5S), were propagated in post-inoculation medium (PI medium) containing DMEM supplemented with 0.3% tryptose phosphate broth (TBP), 0.02% yeast extract, and 10 μg/mL trypsin as described previously [18].

### 2.3. Generation and Recovery of Recombinant iPEDVPT-P5, iPEDVPT-P96, iPEDVPT-P5-96S and iPEDVPT-P96-5S Viruses

The strategy used to recover iPEDVPT-P96 has been described previously [19]. The approach to constructing a cDNA clone of iPEDVPT-P5 was technically identical to that of the iPEDVPT-P96. However, we split the plasmid B into two fragments because the sequence remained toxic to the One Shot™ TOP10 Chemically Competent *E. coli* cells (Invitrogen, Carlsbad, USA) despite propagation in LB broth supplemented with 10% SOC medium and being incubated at 30 °C. To generate chimeric viruses carrying heterologous *spike (S)* genes, namely the iPEDVPT-P5-96S and iPEDVPT-P96-5S, cDNA clones of iPEDVPT-P5 and iPEDVPT-P96, respectively, were used as the backbones. The sequences covering the complete *S* gene of each virus were exchanged without disruption to the remaining genomic structure. Sequence differences in the *S* genes of iPEDVPT-P5 and iPEDVPT-P96 viruses are summarized in Table 1. Each plasmid was digested with corresponding type-IIS restriction enzymes as designated in Figure S1, gel-purified, assembled and phenol-chloroform extracted to generate the full-length cDNAs. The cDNAs were then in vitro transcribed to the full-length RNA transcripts using a mMessage mMachine T7 transcription kit (Ambion, Austin, CA, USA) and immediately electroporated into 800 μL of 10^7^ cells/mL Vero cells in RNase-free phosphate buffered saline (PBS) along with 5 μg of PEDV nucleocapsid (N) transcripts. After electroporation, the cells were allowed to recover in growth medium for approximately 16 h and then maintained in PI-medium until cytopathic effects involved over 90% of cell monolayers. The whole flasks were subjected to one freeze-and-thaw cycle and the rescued viruses were passaged once to generate viral stocks (P1). The viral stocks were titrated on Vero cells in 96-well plates to determine the viral titer (see below).

### 2.4. In Vitro Characterization of Recombinant iPEDVPT-P5, iPEDVPT-P96, iPEDVPT-P5-96S and iPEDVPT-P96-5S Viruses

#### 2.4.1. Immunofluorescence Assay and Syncytia Analysis

Immunofluorescence assay (IFA) was performed to detect PEDV antigens as previously described with modifications [18]. Briefly, Vero cells in 96-well plates (1.75 × 10^4^ cells/well) were infected with the designated P1viruses at a multiplicity of infection (MOI) of 0.005. At 18 h post-infection, cells were fixed with 80% ice-cold acetone, air-dried, and then incubated with an in-house anti-PEDV S antibody, P4B [20], at a dilution of 1:1000 at room temperature (RT) for 1 h (h). After being washed three times with phosphate-buffered saline (PBS), the FITC-conjugated monoclonal goat anti-mouse-IG antibody (BD Pharmingen, San Jose, CA, USA) was applied at a dilution of 1:500 at RT for 1 h. Following the final wash step, the cells were counterstained with mounting medium with 4′,6-diamidino-2-phenylindole (DAPI; Abcam, Cambridge, MA, USA) in the dark for 1 min. Images were visualized and captured using ZOE fluorescent cell imager (Bio-Rad, Hercules, CA, USA). Syncytia analysis were performed concurrently along with IFA by calculating the number of nuclei per syncytium.

#### 2.4.2. Sequence Analysis

**Figure 1:**
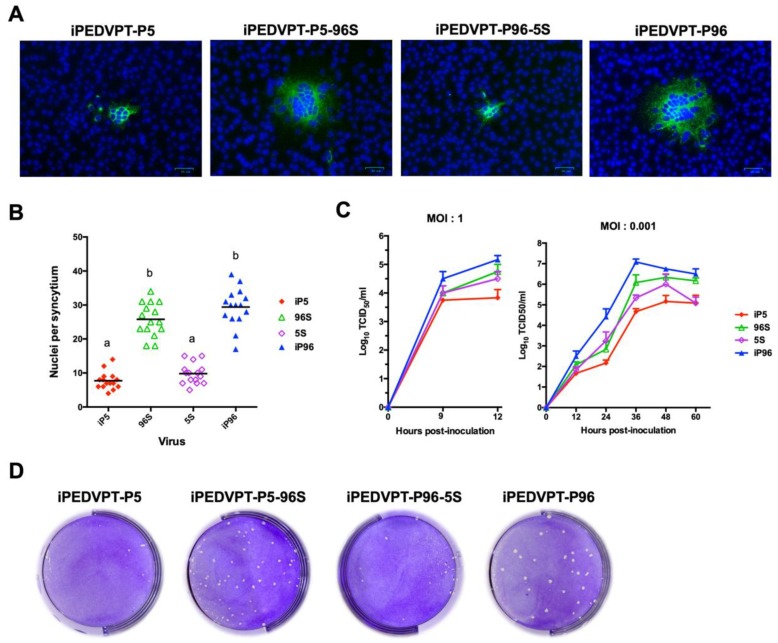
In vitro characterization of the recombinant iPEDVPT-P5, iPEDVPT-P5-96S, iPEDVPT-P96-5S and iPEDVPT-P96 viruses. (**A**) Representative immunofluorescence micrographs of porcine epidemic diarrhea virus (PEDV) syncytia in Vero cells stained with anti-PEDV spike antibody at 18 h post-infection (HPI). Note the recombinant viruses carrying *spike* gene derived from the attenuated iPEDVPT-P96 showed larger syncytia. Scale Bar = 50 μm. (**B**) The number of nuclei per syncytium was quantified and assessed statistically using Kruskal–Wallis test after immunofluorescence staining. Statistical differences (*p* < 0.05) among groups were indicated by different letters. (**C**) Growth kinetics of recombinant viruses in Vero cells after infection at multiplicity of infection (MOI) of 1 and 0.001. Supernatant were collected at indicated time points and the titers were quantified in Vero cells by standard 50% tissue-culture infectious dose (TCID_50_) assay. (**D**) Plaque morphologies in Vero cells of the recombinant viruses. The plates were stained with 1% crystal violet solution after 72 HPI. Note that comparing to the iPEDVPT-P96 and iPEDVPT-P5-96S, the plaques produced by iPEDVPT-P5 virus and iPEDVPT-P96-5S were barely visible macroscopically. iP5: iPEDVPT-P5; 96S: iPEDVPT-P5-96S; 5S, iPEDVPT-P96-5S; iP96, iPEDVPT-P96.

Sequence analysis was conducted as described previously [18,19] and two primer pairs (SF-7: ACTCTCGACTGGACATTC and 2R: CAGACTTCGAGACATCTTTG; 5FR-3: ATTAGAGCGATTCTCCATGAC and 5FR-6: TACACACATTGTGGTGCTATTGAG) targeting the C-terminal end of the *S* gene, which contained both naturally occurred and artificially introduced marker mutations (Figure 1, asterisks and Table 1), as well as the *non-structural protein 15 (nsp 15)* gene, which contained a naturally occurred mutation (G19470T) were used to verify the identities of the four P1 viral stocks and the recombinant viruses shed in feces per group at the time point of peak fecal viral shedding.

#### 2.4.3. Growth Kinetics, Viral Titration and Plaque Assay

Confluent monolayers of Vero cells were seeded onto six-well plates (5 × 10^5^ cells/well) and infected with each virus at a MOI of 1 and 0.001 at 37 °C for 1 h in triplicates. The cells were then washed twice with Dulbecco’s phosphate-buffered saline (DPBS) and maintained in PI medium. The supernatants at indicated time points were collected and proceeded for viral quantification on Vero cells in 96-well plates using the standard 50% tissue-culture infectious dose (TCID_50_) assay. In brief, Vero cells in 96-well plates were washed twice with DPBS and then incubated with a 10-fold serially diluted culture supernatant acquired from the aforementioned six-well plate at 37 °C for 1 h. After absorption, the inoculum was removed and replaced with fresh PI-medium following one wash step. The titers were determined at 72 h post-infection using the Reed–Muench method [21].

Plaque assays were performed as previously described [19] to characterize plaque morphologies. Briefly, after absorption of PEDVs at an MOI of 0.0001, confluent monolayers of Vero cells in six-well plates were washed twice with DPBS and then covered with an overlay of pre-warmed PI medium containing 1% agarose. After solidification of the overlays, the plates were incubated at 37 °C for 72 h to produce distinct plaques. The cells were then fixed in 3.16% neutral-buffered formalin for 1 h before removing the semisolid overlays. The plates were stained with 1% crystal violet in 20% ethanol and distilled water for 1 min. Viral plaques were inspected after washing off the crystal violet solution, rinsing the plates with water, and air-drying at room temperature (RT).

### 2.5. Animal Experiment

Thirty-seven, six-day-old, Large White × Duroc, crossbred, fecal PEDV and TGEV shedding-negative suckling piglets were purchased from a conventional pig farm devoid of G2b PEDV infection history based on the negative result of our long-term surveillance of serum antibody and colostrum against PEDV in this pig farm. These piglets from different sows were fed with artificial milk and were randomly assigned to five groups, acclimated for one day, and then inoculated orally with indicative recombinant viruses at a dose of 2 mL of 0.5 × 10^2^ TCID_50_/mL or PI-medium, respectively. Clinical signs and weight gain were recorded daily. Fecal consistency was monitored daily and scored visually as: 0 = normal, 1 = loose, 2 = semi-fluid, and 3 = watery as previously described. Calculation of average daily weight gain was only performed on piglets that were not humanly euthanized. The formula used for calculation is as follows: Weight gained/ surviving period.

#### 2.5.1. Quantification of PEDV Fecal Viral Shedding

Methods to quantify fecal PEDV viral shedding has been described previously [19]. Briefly, fecal samples collected from rectal swabs were resuspended in DPBS and then subjected to automated nucleic acid extraction using Cador Pathogen 96 QIAcube HT Kit with QIAcube (Qiagen Inc., Hilden, Germany) according to the manufacturer’s instructions. Complementary DNA was synthesized via reverse transcription using QuantiNova™ Reverse Transcription kit (Qiagen Inc., Hilden, Germany) and proceeded to quantitative real-time PCR analysis using the primer-probe set published previously on a CFX96 Thermal Cycler (Bio-Rad, Hercules, CA, USA). The thermal profile comprised an initial denaturation at 95 °C for 2 min and then 45 cycles of 95 °C for 15 s followed by 60 °C for 15 s. The detection limit of the assay was determined by generating standard curves from serial 10-fold dilutions of known amounts of in vitro transcribed RNA followed by reverse transcription and real-time PCR quantification as described above. The detection limit was calculated as 4.8 log_10_ RNA copies per mL.

#### 2.5.2. Histopathology and Immunohistochemistry

At three days post-inoculation, three pigs from each virus-treated group and one pig from mock group were humanely euthanized by electrocution followed by exsanguination for histopathological and immunohistochemical assessments, as described previously [22]. Duodenum, jejunum, ileum, cecum, colon, rectum and mesenteric lymph nodes were collected, formalin-fixed, paraffin-embedded, sectioned at 4 μm, and stained routinely with hematoxylin and eosin (H&E) for morphometric analysis by assessing the ratio of villi height to crypt depth blindly by one veterinary pathologist.

Immunohistochemistry was performed to evaluate the distribution of PEDV antigen. Briefly, formalin-fixed paraffin-embedded tissues were sectioned at 4 μm, deparaffined in xylene, rehydrated in serially diluted ethanol, and proceeded to epitope retrieval with the Trilogy antigen retrieval system (Cell Marque, Rocklin, CA, USA). After being washed three times with Tris-buffered saline plus 0.1% Tween 20 (TBST), tissue slides were treated with 3% hydrogen peroxidase (KYB, New Taipei City, Taiwan) and 10% normal goat serum (Dako, Carpinteria, CA, USA) to block the endogenous peroxidase activity and non-specific signals, respectively. For antigen detection, an in-house anti-PEDV N antibody, DE-1, at a dilution of 1:1000 in 10% normal goat serum was applied to the slides for 1 h at RT followed by three times wash with TBST. The first antibodies were then captured using the polyclonal anti-rabbit/mouse immunoglobulin, EnVision-DAB^+^ system (Agilent Technologies, Santa Clara, CA, USA) at RT for 1 h and color was developed afterward with 3, 3′-diaminobenzidine (DAB) chromogen (Agilent Technologies, Santa Clara, CA, USA). The slides were counterstained with hematoxylin (MUTO, Tokyo, Japan), mounted in Entellan (Merck, Darmstadt, Germany) and cover slipped. Positive signals were visualized under an inverted light microscope (Nikon, Tokyo, Japan).

### 2.6. Statistical Analysis

All values were expressed as the mean standard ± deviation (SD). Comparison of syncytia size and villous height to crypt depth (VH:CD) ratio were analyzed using statistical software GraphPad Prism 6.0 (GraphPad Prism Inc., San Diego, CA, USA). Variables were compared using the non-parametrical Kruskal–Wallis test; *p* < 0.05 was considered statistically significant.

## 3. Results

### 3.1. Recovery of recombinant PEDVs

We have recently developed a reverse genetic platform of PEDV and have successfully generated a recombinant virus, iPEDVPT-P96, which is phenotypically comparable to its parental attenuated Taiwan PEDV-Pintung 52 strain, PEDVPT-P96 [19]. To study the role of the *S* gene in the attenuation mechanism of PEDVPT-P96, we further generated the recombinant, highly virulent Taiwan PEDVPT 52 strain, iPEDVPT-P5, and two chimeric viruses (iPEDVPT-P5-96S and iPEDVPT-P96-5S) by replacing the complete sequence of the *S* gene reciprocally (Figure S1). In general, the approach to rescue the new recombinant viruses were virtually the same as published previously [19]. However, for the generation of iPEDVPT-P5 and iPEDVPT-P5-96S, we split the plasmid B to two fragments at nucleotide position of 9654 according to Hou et al. [23] in order to compensate for the instability caused by the toxic sequences. The in vitro and in vivo properties of iPEDVPT-P5 were confirmed similarly to those of the parental PEDVPT-P5 strain in a seven-day-old conventional piglet model.

At 24–48 h post-electroporation, typical PEDV cytopathic effects of giant syncytia were observed for all recombinant viruses. Viral stocks were prepared by passaging viruses in Vero cells one additional time (P1), and the presence of PEDV was confirmed by detection of PEDV S protein by immunofluorescence assay (Figure 1A) and sequence analyses.

### 3.2. In Vitro Characterization of Recombinant PEDVs

The in vitro characteristics of the recombinant viruses were compared by evaluation of the size of syncytia, growth kinetics in Vero cells, and plaque morphologies. All recombinant viruses induced formation of syncytia by 18 h post-infection. Viruses carrying the identical *S* gene exhibited similar fusogenic ability as suggested by the comparable number of nuclei per syncytium; representative micrographs used to count the number of nuclei in syncytia are shown in Figure 1A. Remarkably, viruses carrying *S* gene derived from iPEDVPT-P96, induced 3.5 to 4 times larger syncytia than the other two viruses containing *S* gene derived from iPEDVPT-P5 (Figure 1B).

Replication of all recombinant viruses in Vero cells were examined by performing both one-step and multistep growth kinetics at a multiplicity of infection (MOI) of 1 and 0.001, respectively. Interestingly, although replacement of the *S* gene resulted in an alteration of the replication kinetics and efficiency, leading to similar growth curve patterns between viruses carrying the identical *S* gene sequence, it did not fully reverse the capability of virus yield, at least in Vero cells (Figure 1C).

We observed that plaque morphologies of the four recombinant viruses correlated to the results of syncytia size. At 72 h post-infection, both iPEDVPT-P5 and iPEDVPT-P96-5S induced barely visible viral plaques in Vero cells macroscopically, whereas iPEDVPT-P96 and iPEDVPT-5-96S generated distinct and comparable viral plaques with the plaque size of iPEDVPT-P5-96S being slightly smaller than that of the iPEDVPT-P96 (Figure 1D). These data suggested that the exchange of the *S* gene between iPEDVPT-P5 and iPEDVPT-P96 affected the fusogenicity and, to a lesser extent, replication kinetics in Vero cells.

### 3.3. Investigation of the Role of Spike Gene on the Pathogenicity of PEDVPT 52 strain

**Figure 2:**
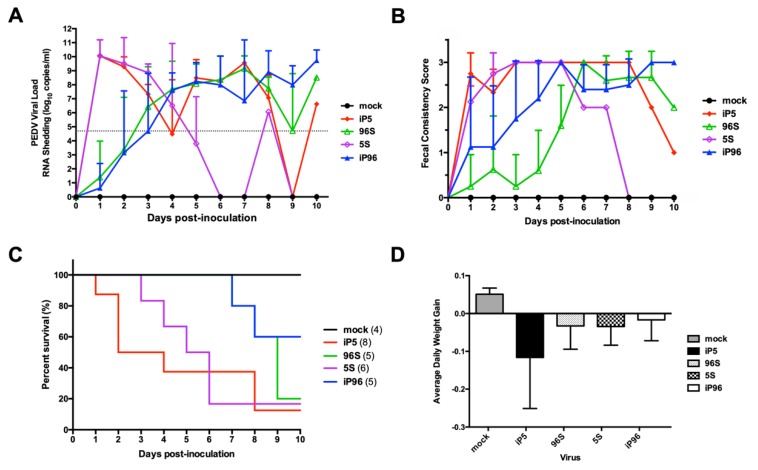
In vivo characterization of the recombinant iPEDVPT-P5, iPEDVPT-P5-96S, iPEDVPT-P96-5S and iPEDVPT-P96 viruses in conventional seven-day-old piglets. (**A**) Mean value of RNA copies in feces in conventional seven-day-old piglets after inoculation with recombinant viruses or PI-medium through the study. The dash line indicated the detection limit of the real-time PCR (log^4.8^ RNA copies per mL). Error bars represented standard deviation. (**B**) Mean value of fecal consistency graded daily in piglets after inoculation with recombinant viruses or PI-medium through the study. Error bars represented standard deviation. (**C**) Survival curve of piglets after inoculation with recombinant viruses or PI-medium through the study. (**D**) Mean value of average daily weight gain in different treated groups. Calculation of average daily weight gain was only performed on piglets that were not euthanized. Below is the formula used for calculation: Weight gained surviving period. Error bars represented standard deviation. Mock: PI-medium; iP5: iPEDVPT-P5; 96S: iPEDVPT-P5-96S; 5S, iPEDVPT-P96-5S; iP96, iPEDVPT-P96.

**Figure 3:**
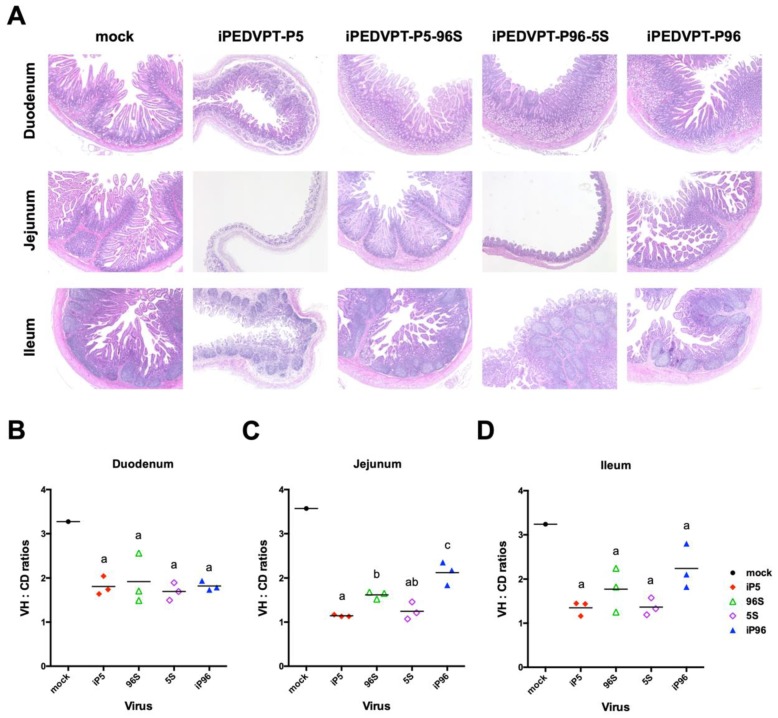
Histopathological evaluation and morphometric analysis. (**A**) Representative micrographs (magnification, × 40) demonstrating variable degrees of villous atrophy at duodenum, jejunum and ileum from piglets inoculated with different recombinant viruses or post-inoculation (PI)-medium. Tissues were collected at 3 d post-inoculation and processed routinely for slide preparation for hematoxylin and eosin staining. (**B**–**D**) Villous height to crypt depth (VH:CD) ratios in the duodenum (**B**), jejunum (**C**), and ileum (**D**) for piglets at 3 DPI after inoculation with different recombinant viruses or PI-medium. Ten villi of each intestinal segment were examined in each piglet and each symbol represented the mean value of VH:CD ratio in an individual piglet. Statistical differences (*p* < 0.05) among virus-treated groups were compared using the Kruskal–Wallis test. Different letters indicate statistical significance (*p* < 0.05). Mock: PI-medium; iP5: iPEDVPT-P5; 96S: iPEDVPT-P5-96S; 5S, iPEDVPT-P96-5S; iP96, iPEDVPT-P96.

To evaluate the pathogenicity related to *S* gene replacement in PEDVPT 52 strain, we orally inoculated 2 mL of 5 × 10^2^ TCID_50_/mL of iPEDVPT-P5, iPEDVPT-P5-96S, iPEDVPT-P96, iPEDVPT-P96-5S viruses and with PI medium in seven-day-old crossbred piglets assigned in the corresponding groups. Parameters used to assess the pathogenicity of different recombinant viruses were summarized in Table 2 and shown in Figure 2. The target sequences of the recombinant viruses at the time point of peak fecal viral shedding in each pig was confirmed identical to the original inoculum. No viral RNA shedding, diarrhea or mortality was detected in the mock-treated group during the entire experimental course. Piglets inoculated with recombinant viruses carrying the *S* gene derived from the highly virulent iPEDVPT-P5, namely the iPEDVPT-P5 itself and iPEDVPT-P96-5S, had an early onset of clinical symptoms including diarrhea, anorexia and decreased activity, and peak viral shedding at 1 d post-inoculation (DPI). At 3 DPI, piglets inoculated with iPEDVPT-P5 (*n* = 3) and iPEDVPT-P96-5S (*n* = 3) both exhibited extensive PEDV-induced villous blunting and atrophy in the jejunum and, to a lesser extent, in the ileum; histological changes in the duodenum were not obvious (Figure 3). Mortality rates of both groups were comparable, reaching 50% at 2 (iPEDVPT-P5 group) and 5 (iPEDVPT-P96-5S group) DPI and ultimately exceeded 80% by 10 DPI. 

In comparison, inoculation with iPEDVPT-P5-96S and iPEDVPT-P96 viruses containing the *S* gene derived from the attenuated iPEDVPT-P96, induced a delayed onset of clinical symptoms and peak viral shedding. Histological evaluation of piglets inoculated with both viruses (iPEDVPT-P96, *n* = 3; iPEDVPT-P5-96S, *n* = 3) at 3 DPI revealed a much milder degree of villous atrophy in the jejunum with conspicuous villous hyperplasia compared to the other two groups. Notably, iPEDVPT-P5-96S-treated piglets exhibited statistically significant severer villous atrophy in jejunum than iPEDVPT-P96-treated piglets (Figure 3). Despite the similar pattern and severity of viral shedding and clinical symptoms, inoculation with iPEDVPT-P5-96S eventually resulted in a higher mortality rate (40% in iPEDVPT-P96 and 80 % and iPEDVPT-P5-96S) and lower average daily weight gain by 10 DPI than those upon inoculation with iPEDVPT-P96. These data suggested that iPEDVPT-P96 fully regained virulence by replacement of the *S* gene from the virulent PEDVPT 52 strains and the complementary approach can only partially reduce the virulence of the iPEDVPT-P5.

**Figure 4:**
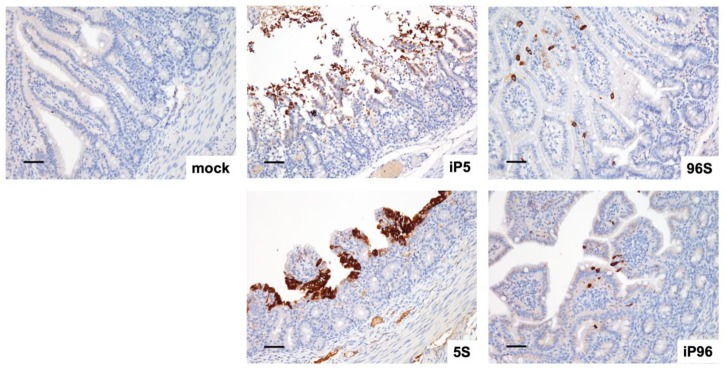
Immunohistochemical staining of PEDV nucleocapsid (N) at jejunum of piglets inoculated with different recombinant viruses or PI-medium at 3 d post-inoculation (DPI). The representative micrographs were taken and shown above. Scale Bar = 50 μm.

In agreement with the previous study, immunohistochemistry using anti-PEDV nucleocapsid monoclonal antibody demonstrated positive signals predominantly in the jejunal and ileal enterocytes at the top of villi (Figure 4) but occasionally within the mesenteric lymph nodes. No difference in the viral distribution was identified among the recombinant viruses.

## 4. Discussion

Porcine epidemic diarrhea (PED) to date remains a colossal burden to the global swine industries owing to the lack of successful vaccine in the field [12,13]. Moreover, other emerging and re-emerging swine enteric coronaviruses, including porcine deltacoronavirus (PDCoV) [24], and swine acute diarrhea syndrome coronavirus (SADS-CoV) [25], further complicate the field condition by affecting diagnostic accuracy and increasing the risk of viral recombination [26]. Hence, there is still a pressing need to develop a safe and effective vaccine, particularly the LAV, to mount this disastrous disease. The reverse genetics system is a powerful and widely used tool to study viral pathogenesis and novel LAV design by active modification of genes of interests. Previous studies using reverse genetics on other coronaviruses have identified many virulent/attenuating determinants that might be shared among different genus of coronaviruses [12]. Nevertheless, direct evidences of attenuation due to harboring those mutated determinant(s) in pigs are still limited in PEDV [16,23,27]. In the present study, to investigate the role of the *S* gene in the attenuation process of the PEDVPT 52 strain, we generated four infectious cDNA clones of G2b PEDV, including the parental virulent iPEDVPT-P5, attenuated iPEDVPT-P96, as well as two chimeric viruses (iPEDVPT-P5-96S and iPEDVPT-P96-5S) with exchanged *S* gene. We found that the iPEDVPT-P96 virus fully regained the virulence after the exchange of *S* gene derived from the highly virulent iPEDVPT-P5 virus, showing comparable patterns of viral shedding, diarrhea and histopathological changes. Our data confirmed that the *S* gene is the primary attenuating determinant of the iPEDVPT-P96 virus and the genetic backbone other than the *S* gene involving the attenuation of the iPEDVPT-P96 might be of less importance. However, the iPEDVPT-P5-96S still exhibited partial virulence resulting in severer villous attenuation and higher mortality rate compared to those caused by the iPEDVPT-P96 virus. This result supports that the virulence of PEDV might be a multigenic event. 

In the present study, the first pair of cDNA clones of a virulent G2b PEDV and its derived attenuated strain were generated. Since these two viruses were closely related to each other, this established platform would allow for easier manipulate in studying the effect of single nucleotide polymorphism for identifying potential virulent/attenuating determinate(s). In the present study, we demonstrated that reversion of virulence or attenuation occurred after the *S* gene was reciprocally replaced. However, this finding is in disagreement with previously published results by Wang et al. [17], indicating that there was an absence of virulence reversion after *S* gene substitution between the highly virulent G2b strain, BJ2011C, and the avirulent G1a strain, CHM2013. There could be a few reasons for the discrepancy between our observations that of previous studies. First, the autonomy of the viruses is different. Viral attenuation in serial cell-culture or animal passage is a progressive process that involves a series of gene mutations and the subsequent alternated cooperative interplay between gene products and mechanisms influencing cell-virus interaction. Although the patterns of attenuating mutation are similar among G2b PEDV strains, the asynchronous mutations between different PEDV strains could lead to loss of the cooperative or complementary function(s) for other gene products. In the previous study, the used viral strains belonged to different genogroups [17,28]. Efficient viral assembly requires proper signaling and interaction between each structural protein. Since the BJ2011C virus belonged to genogroup 2b, we speculated that the reason a singular *S* gene exchange failed to reverse the virulence of the avirulent G1a CHM2013 strain might be due to, at least in part, the suboptimal cooperation between structural proteins in terms of PEDV morphogenesis. Second, the degree of attenuation differed between the CHM2013 virus and PEDVPT-P96 virus. Comparing to the avirulent CHM2013 virus that induced no detectable viral shedding in two-day-old piglets, iPEDVPT-P96 virus retained some levels of virulence as it caused viral shedding, observable clinical symptoms, and even mortality in the seven-day-old conventional piglets. Besides the *S* gene [17,28], the abolishment of the function of non-structural proteins (NSPs) alone has been demonstrated to attenuate highly virulent PEDVs by disrupting the antagonistic ability of host interferons (IFN) [16,27]. That means, when the function of certain NSP was abated, PEDVs lose its virulence, regardless of the *S* gene they carry. Therefore, variations of NSPs in the CHM2013 virus might have a critical effect on its virulence, and the effect might be stronger than that contributed by the *S* gene derived from the highly virulent BJ2011C virus. Similarly, we also speculated that the IFN-suppressive function provided by NSP(s) might also contribute to virulent differences between the iPEDVPT-P5-96S and iPEDVPT-P96 since nine mutations were identified in NSPs in the iPEDVPT-P96 as compared with that of PEDVPT-P5 [18]. Regardless, further studies are needed to clarify the hypotheses mentioned above.

Sequence comparison between the autologous highly virulent PEDVPT-P5 and attenuated PEDVPT-P96 revealed several amino acid substitutions [18], especially in the *S* gene (see Table 1). Consistent with other studies, these mutations chiefly accumulated in the S2 domain, presumably because of adaptation to Vero cells [12,29]. In the present study, the enhanced fusogenic ability in vitro was distinctly ascribed to the *S* gene, more specifically the S2 domain, derived from iPEDVPT-P96 and presumably represented the evolutionary process to increase viral progeny since Vero cells are not of swine-origin but readily susceptible to PEDV infection. Animal experiments with seven-day-old piglets revealed that the exchange of the *S* gene from the iPEDVPT-P96 resulted in a delayed onset of peak viral shedding, diarrhea, and milder villous atrophy when examined histologically at 3 d post-inoculation. Additionally, in contrast to the findings published by Wang et al. [17], Suzuki et al. [30] reported that replacement of the entire *S* gene or S1 sequence from a highly virulent OKN-1/JPN/2013 American type PEDV strain enabled the attenuated rPEDVGFP-CV777 to acquire virulence in piglets. In iPEDVPT-P96 virus, however, only two amino acid substitutions were identified in the S1^0^ (C144T) and S1^B^ (T554C) domains compared to the iPEDVPT-P5 virus. For PEDV, S1^0^ and S1^B^ domains are known to contain sialic acid-binding and receptor-binding domains, respectively; thus, are crucial for viral entry [31]. Although Hou et al. [23] previously showed that deletion of 197 amino acids in S1^0^ domain of PEDV resulted in attenuation in piglets, a more specific epitope has yet to be identified. Therefore, we are curious about whether only these two mutations in iPEDVPT-P96 can attenuate the highly virulent iPEDVPT-P5 virus or if the S2 domain must be primarily accounted for the attenuation. Future studies will be conducted to elucidate these questions.

In this study, the first pair of cDNA clones for a virulent G2b PEDV, and its derived attenuated strain were generated, allowing us to use both gain-of-function and lose-of-function approaches to studying the role of the *S* gene in PEDV pathogenesis. We confirmed that the *S* gene is a crucial virulent/attenuating determinant for the iPEDVPT-P96, but its importance varies among different PEDV strains. Thus, other studies’ results alongside our results will provide valuable information for the future generation of novel chimeric or multivalent vaccines.

## Figures and Tables

**Table 1:** Nucleotide and amino acid differences of spike gene between the virulent iPEDVPT-P5 and attenuated iPEDVPT-P96 Viruses.

Position in Spike Gene			Nucleotide		Amino Acid	
Nucleotide	Amino Acid	Domain	iPEDVPT-P5	iPEDVPT-P96	iPEDVPT-P5	iPEDVPT-P96
431	144	S1^0^	C	T	T	I
1661	554	S1^B^	T	C	F	S
2659	887	S2	A	C	S	R
2902	968	S2	T	G	S	A
3062	1021	S2 HR1 ^†^	T	G	I	S
3077	1026	S2 HR1 ^†^	A	G	K	R
3755	1252	S2	T	G	L	R
4061	1354	S2 CT ^‡^	G	T	C	F
4073	1358	S2 CT ^‡^	G	T	C	F
4134	1378	S2 CT ^‡^	A	T	E	D

^†^ HR1: heptad repeat 1; ^‡^ CT: cytoplasmic tail.

**Table 2:** Summary of clinical observations used for evaluating the pathogenicity of recombinant viruses.

Group	Mortality Rate (%)	Peak Viral Shedding (DPI)	Peak Viral Shedding (log_10_ copies/mL)	Onset of Diarrhea (DPI) ^†^	Mean Average Daily Weight Gain ^‡^
**iPEDVPT-P5**	87.5 (7/8)	1	10.06 ± 1.14	1.00 ± 0.0	−0.116 ± 0.135
**iPEDVPT-P5-96S**	80.0 (4/5)	7	9.15 ± 0/91	3.83 ± 1.47	−0.033 ± 0.062
**iPEDVPT-P96-5S**	83.4 (5/6)	1	8.20 ± 1.70	1.00 ± 0.0	−0.035 ± 0.049
**iPEDVPT-P96**	40.0 (2/5)	10	9.74 ± 0.74	2.38 ± 1.51	−0.016 ± 0.055
**Mock**	0.0 (4/4)	ND	ND	ND	0.051 ± 0.016

^†^ Onset of diarrhea was defined as day post-inoculation when loose stools scored as two or above were recorded. ^‡^ Average daily weight gain (AVG) was calculated by dividing weight gained by survival days; mean value was calculated as dividing the sum of AVG by the number of piglets in the indicated groups that were not humanely euthanized.

## Supplementary Materials

The following are available online at https://www.mdpi.com/1999-4915/12/1/41/s1, Figure S1: Schematic diagram of cloning strategy to generate recombinant viruses.
