# Supplementary material for: Investigation of the Role of the Spike Protein in Reversing the Virulence of the Highly Virulent Taiwan Porcine Epidemic Diarrhea Virus Pintung 52 Strains and Its Attenuated Counterpart
Source: Viruses. 2019 Dec 30;12(1):41. doi: 10.3390/v12010041 (PMC7019868; doi:10.3390/v12010041)
Supplement: Supplementary file 1 [file viruses-12-00041-s001.pdf]

Supplementary Data

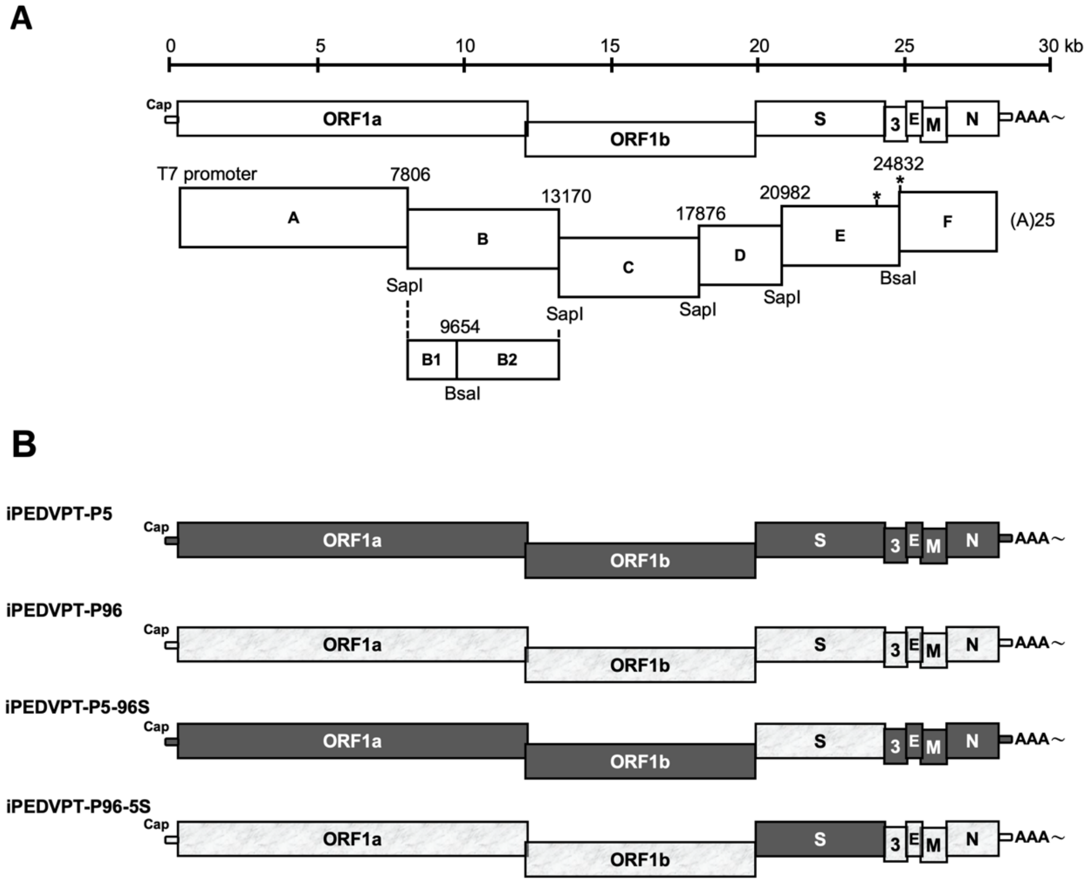

Figure S1. **Schematic diagram of cloning strategy to generate recombinant viruses.** (A) The organization of the complete PEDV genome and the DNA fragments used to generate full-length cDNA clones. The nucleotide sites joined with specific restriction enzymes were shown and labelled. The representative locations wherein marker mutations were introduced were marked with asterisks. (B) Diagram of genome structure of the recombinant viruses carrying the original or exchanged spike gene. ORF, open reading frame; S, spike; 3, ORF3; envelop; M, membrane; N, nucleocapsid. The genes derived from the virulence iPEDVPT-P5 backbone were shown as dark boxes whereas those derived from the attenuated iPEDVPT-P96 backbone were depicted as gray boxes.
